# Oral Dysbiosis and Autoimmunity: From Local Periodontal Responses to an Imbalanced Systemic Immunity. A Review

**DOI:** 10.3389/fimmu.2020.591255

**Published:** 2020-12-08

**Authors:** Lina J. Suárez, Hernan Garzón, Silie Arboleda, Adriana Rodríguez

**Affiliations:** ^1^ Departamento de Ciencias Básicas y Medicina Oral, Universidad Nacional de Colombia, Bogotá, Colombia; ^2^ Grupo de Investigación en Salud Oral, Universidad Antonio Nariño, Bogotá, Colombia; ^3^ Unidad de Investigación en Epidemiologia Clínica Oral (UNIECLO), Universidad El Bosque, Bogotá, Colombia; ^4^ Centro de Investigaciones Odontológicas, Pontificia Universidad Javeriana, Bogotá, Colombia

**Keywords:** autoimmunity, autoantigens, autoantibodies, oral, dysbiosis, microbiome, periodontitis, *Porphyromonas gingivalis*

## Abstract

The current paradigm of onset and progression of periodontitis includes oral dysbiosis directed by inflammophilic bacteria, leading to altered resolution of inflammation and lack of regulation of the inflammatory responses. In the construction of explanatory models of the etiopathogenesis of periodontal disease, autoimmune mechanisms were among the first to be explored and historically, for more than five decades, they have been described in an isolated manner as part of the tissue damage process observed in periodontitis, however direct participation of these mechanisms in the tissue damage is still controversial. Autoimmunity is affected by genetic and environmental factors, leading to an imbalance between the effector and regulatory responses, mostly associated with failed resolution mechanisms. However, dysbiosis/infection and chronic inflammation could trigger autoimmunity by several mechanisms including bystander activation, dysregulation of toll-like receptors, amplification of autoimmunity by cytokines, epitope spreading, autoantigens complementarity, autoantigens overproduction, microbial translocation, molecular mimicry, superantigens, and activation or inhibition of receptors related to autoimmunity by microorganisms. Even though autoreactivity in periodontitis is biologically plausible, the associated mechanisms could be related to non-pathologic responses which could even explain non-recognized physiological functions. In this review we shall discuss from a descriptive point of view, the autoimmune mechanisms related to periodontitis physio-pathogenesis and the participation of oral dysbiosis on local periodontal autoimmune responses as well as on different systemic inflammatory diseases.

## Introduction

The immune system has an uneasy relationship with the environment (1), being a mobile network of cells with emergent properties derived from dynamic cellular interactions that have evolved to guard the body against attack (2). Under normal conditions, the immune system exhibits tolerance, and must be able to distinguish self from non-self and harmless non-self from dangerous non-self (1). When self-tolerance is disrupted, autoimmunity will arise (3). The alteration in the activation of almost any immune process in a susceptible host could trigger immune dysregulation and autoimmunity. This fact is more clearly understood from the analysis of primary immunodeficiency disorders, initially described in patients who had severe or recurrent infections from which the contradiction arises that individuals who were unable to respond against foreign antigens, did so against their own antigens. Immunodeficiencies in which the flaws are not in the effector response but in the immune regulatory mechanisms, have been called immune dysregulation disorders (4, 5). Autoimmunity and immunodeficiencies are today considered two sides of the same coin, being conditions that are not mutually exclusive and that are both linked to alterations in signaling mechanisms and immune regulation; genetic defects have been described not only as the cause of primary immunodeficiency syndromes, but also as factors that predispose to infections and lead to dysregulation and autoimmunity (6).

The host-microbiome relationship must be understood today far beyond the concept of disease. The microbiome is related to multiple functions in the host, including the control of vascularization at the intestinal level (7) and the functioning of the central nervous system affecting the production of soluble mediators (8); but without a doubt, the most recognized role of the commensal microbiome in maintaining healthy homeostasis is its immunostimulatory effect. This includes the recruitment of mucosal immune cells, the generation and maturation of organized lymphoid tissues (9), and the stimulation of protective functions of epithelial cells such as mucus formation and production of antimicrobial peptides (10).

Given the recognition of the role of commensal microbiota in the development of immunity and the regulation of the immune response against pathogens, it is not unreasonable to think that this microbiota is also capable of regulating/controlling autoimmune responses. The participation of microorganisms in the occurrence of autoimmunity is being widely supported, as key actors in the loss of microbiome-host homeostasis.

Periodontal disease was recognized for decades as an infectious disease, but more than half a century ago, relationships began to be established with the host’s response to infecting microorganisms, as a determining part of its pathogenesis. In 1965, Brandtzaeg and Kraus (11) made an approach to the immunological mechanisms in periodontal disease, describing for the first time the autoimmune basis in the pathogenesis of periodontitis. They described the presence of anti-collagen antibodies produced by plasma cells in the periodontal tissue of patients with periodontitis. From this, the possible development of an autoimmune process was considered, based on the destructive consequences of this disease in the alveolar bone, conceived at that time as a localized hypersensitivity reaction that involved the formation of immune complexes in the affected periodontal tissues (12).

**Figure 1:**
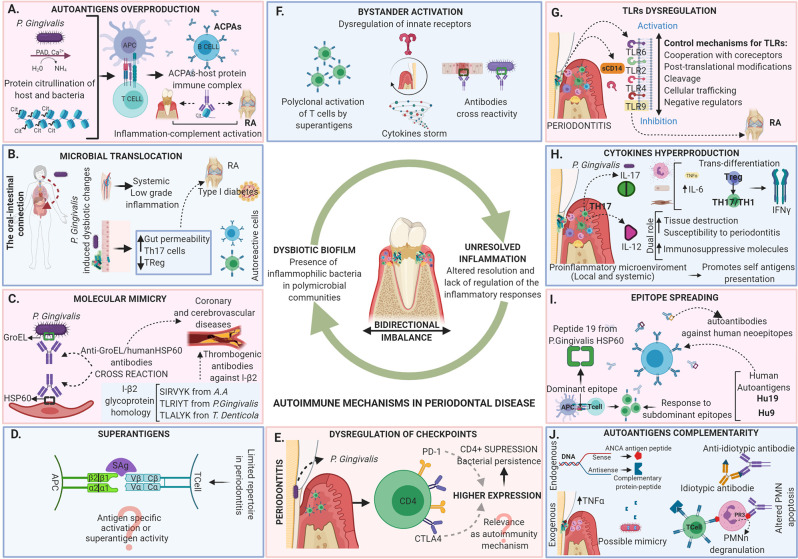
Autoimmune mechanisms in periodontal disease. Periodontitis is today described as the result of dysbiosis of the oral microbiota guided by inflammophilic bacteria, leading to an altered resolution of inflammation and a lack of regulation of the inflammatory response. The tissue damage in periodontitis is directly related to the activation of inflammatory mechanisms by the host that are in a bidirectional imbalance with the microbiome. The dysregulation leads to the appearance of local and systemic autoimmune responses mediated by different mechanisms including **(A)** Autoantigens overproduction, **(B)** Microbial translocation, **(C)** Molecular mimicry, **(D)** Superantigens, **(E)** Dysregulation of checkpoints, **(F)** Bystander activation, **(G)** TLRs dysregulation, **(H)** Cytokines hyperproduction, **(I)** Epitope spreading and **(J)** Autoantigens complementarity. The evidence of the activation of the diverse autoimmune mechanisms in the periodontal tissues does not necessarily indicate that they are related to local tissue damage, although they may be related to the development of autoimmune responses at the systemic level associated with the occurrence of multiple diseases.

As described not even a decade ago, today we understand periodontitis as the result of dysbiosis of the oral microbiota guided by inflammophilic bacteria, leading to an altered resolution of inflammation and lack of regulation of the inflammatory responses (13). The damage is directly related to the activation of inflammatory mechanisms by the host, however not fully elucidated (e.g. the role of autoimmunity in periodontitis pathogenesis) (**Figure 1**).

This review aims to summarize the host and oral microbiome changes involved in the physio-pathogenesis of periodontitis that have been described as contributing factors in local and systemic auto-immune responses leading to disease, as well as to analyze them beyond tissue damage from the physiological activation of the immune system.

### Microbiome-Host Relationship in Autoimmune Responses

Autoimmune processes are the result of a failure in the immune mechanisms of self-tolerance towards components of different nature (proteins, receptors, tissue, etc.) where the microbiota has frequently been involved. The mechanisms by which microorganisms participate in the control of autoimmunity are still unclear, but several approaches have been described. One of the most important, starts from the development of tolerance to infection (14), where resident microorganisms play a very important role by hindering, and even avoiding colonization of their habitat by exercising colonization resistance (15).

It has been observed in animals and humans with genetic predisposition, that the microbiota provides signals that induce antimicrobial effectors that are neutralized by inhibitory microbial signals, establishing as a result, a homeostatic relationship with the host (14). If a specific microorganism’s line expands, this blocks the development of autoimmunity and improves their own chances of staying in that expanded state suppressing the host’s adaptive and inflammatory responses (16). This hypothesis is known as the “specific lineage hypothesis”, in which autoimmunity is “turned off” as a side effect. It could also happen that there is a “signal balance” where, while the host response is balanced against commensals, their effort to reduce it does not affect the development of the disease but the host’s inability to control the microbiota correctly, resulting in the dominance of negative signals provided by microorganisms and the concomitant reduction of autoimmunity (17).

Although the microbiome is strongly involved in the maintenance of health and apparently in the control of autoimmunity, on the other hand, there is sufficient evidence in both humans (18, 19) and animals (20, 21) that supports the role of microorganisms in the occurrence of autoimmune responses. An expansion of pathobionts has recently been found in patients with autoimmune diseases and in animal models of autoimmunity (22).

#### Microbiome and Oral Dysbiosis

The human microbiome is made up of all the microorganisms that live in the human body occupying multiple systems. As the name implies, they are communities -biomes- of microorganisms with very complex interactions with each other and with the host (23). The composition of the human microbiome is highly influenced by changes in the microenvironment. The microflora can be transitory, permanent, pathogenic or non-pathogenic, which has been evolvingly adapted to humans, these being the true commensal microorganisms. Of the latter, some have the ability to modulate the immune response without producing changes in homeostasis known as autobionts, while others are transient pathogens or pathobionts (24) which are not normally pathogenic *per se*, but can trigger immune-inflammatory processes, including autoimmunity in genetically susceptible individuals (22).

The change in the characteristic microorganism communities of a particular microenvironment is known as dysbiosis, also called dysbacteriosis. Bacterial translocation, related to the pathogenesis of multiple diseases, could be better described as atopobiosis, which is the appearance of microorganisms that are characteristic of a certain microenvironment in the “wrong” place. Atopobiosis can occur by multiple ways (25, 26), and is perhaps one of the most relevant mechanisms in the participation of the oral microbiota in multiple conditions and diseases.

It has been demonstrated that microorganisms that are in low numbers in the metagenome can be critical in the community for carrying out essential metabolic activities (18). The mouth of a healthy individual may be colonized by about 100-200 species of the more than 700 oral bacteria identified (27), with inter-individual variations resulting from the environment, genetics, age, and lifestyles (28, 29). The presence of certain mechanisms could prevent dysbiotic changes in the community even in the presence of disease triggers (e.g. greater production of nitric oxide) (30) and their interrelation with the host is then a determining factor in the health-disease process. In fact, homeostasis is triggered by health maintenance mechanisms contributing to resilience especially in tolerant individuals. These mechanisms may be dependent on the microorganisms but also on the host marked by genetic and epigenetic differences, and their identification could lead to the development of new therapeutic strategies aimed at promoting health rather than reducing disease (31).

In the oral cavity and other systems exposed to the external environment, the immune system is constantly exposed to signals that go beyond the commensal-epithelial interaction, including constant damage to tissues resulting from chewing, and antigens from both food and airborne particles. This phenomenon participates in the training of the system by mechanisms that are not entirely clear (32); but what is clear is that the breakdown of the balance of these local responses is related to susceptibility to certain tissue-specific diseases, periodontitis being the prototype, and whose pathogenesis reflects the activation of local and systemic inflammatory processes (including manifestations of autoimmunity), indicating the occurrence of dysregulation of the immune system at this level (33).

Thus, the conception of periodontal disease has changed, relating it now less to a specific bacteria, but more to heterotypic (polymicrobial) groups of microorganisms resulting in dysbiotic communities that alter tissue homeostasis and normal immune responses (34, 35). It has been described that there are community members, specifically *P. gingivalis* (a suggested keystone pathogen in animal models), that even in low numbers (<1%), can increase the virulence of the entire community (36); communication between it and normally commensal microorganisms (accessory pathogens) facilitate synergy and the transition to pathogenicity, allowing the continued development of the dysbiotic community and stimulating the inflammatory response, in which misdirected responses that contribute to tissue destruction are mixed and shape a modified microbiota with “inflammophilic” characteristics, that uses nutrients derived from tissue damage for its survival. The virulence of the community rises as a consequence of the subversion of specific components of the immune response, and thus, this dysbiotic community increases causing additional damage and greater alteration to the homeostasis of the system, mediated mainly by pathobionts (*Filifactor alocis, Peptostreptococcus stomatis*, species of the genus *Prevotella, Megasphaera, Selenomonas*, and *Desulfobulbus*), usually underestimated in periodontitis, but with an apparent better correlation with the disease (37, 38).

The virulence of *P. gingivalis* becomes important when a synergistic microbial community is formed in a susceptible host, indicating that both are necessary for the expression of the microorganism pathogenicity (39). However, other species might be equally or even more active in the process that leads from periodontal health to disease and should be investigated. Besides, it should be noted that the “keystone pathogenesis” itself has yet to be demonstrated in humans (34).

The dysbiosis is closely related to periodontitis, and the relationship between the subgingival microbiome and the immune and inflammatory response seems to be important in the pathogenesis of the disease. The evidence has allowed to hypothesize that microbiome and inflammation in periodontal health are in a bidirectional balance and in periodontitis in a bidirectional imbalance, which seems to be common in other diseases such as sepsis, inflammatory bowel disease, etc (22) (**Figure 1**).

The autoimmunity mechanisms linked to dysbiosis are being actively studied, always having as a basis conditions where the end point is the destruction of the cells of the target tissues by self-reactive cells or autoantibodies, and that involve the participation of microorganisms’ dependent mechanisms of the immune system, both innate and adaptive. Understanding that the microbiome-host interrelation is responsible for the occurrence of tissue damage in dysbiosis related infectious diseases, we grouped the autoimmunity mechanisms into those led by the microorganisms and those where the baton is carried by the immune system, and from the evidence will be related with the autoimmune responses observed in tissue damage/regulation in periodontitis and periodontitis linked systemic diseases.

### Microbial-Dependent Mechanisms of Autoimmunity

#### Autoantigens Overproduction

**Figure 2:**
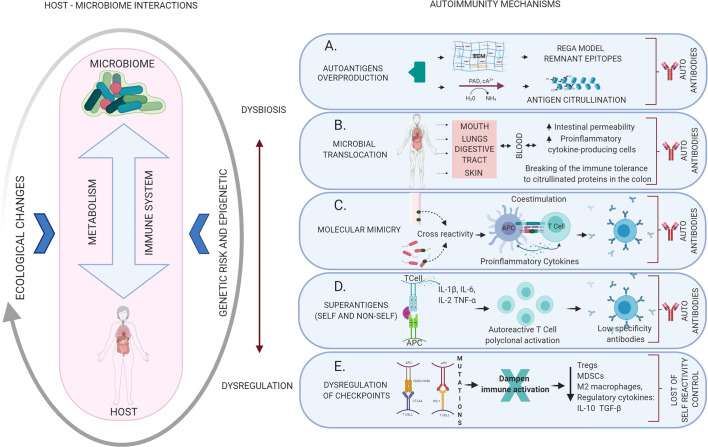
Mechanisms of autoimmunity triggered by dysbiotic changes and dysregulation of the immune system mediated by altered microbiome-host relationships. The host-microbiome interaction may be influenced by several ecological changes (eating habits, aging, infection, among others) that may generate dysbiosis with the consequent activation of different local or systemic autoimmune mechanisms. **(A)** Autoantigens overproduction: the result of the breakdown of the extracellular matrix creating “remnant epitopes” that act as autoantigens or because of enzymatic modification of antigens (citrullination). **(B)** Microbial translocation: bacteria, their products or even products of immune activation against them, can migrate through the bloodstream to different organs leading to an alteration of the local inflammatory response and the production of autoantibodies. **(C)** Molecular mimicry: cross immune response against self-antigens induced by microbial epitopes similar in composition or structure. **(D)** Superantigens: Self or non-self-superantigens interact with the Vβ variable region of the TCR activating and causing T cell expansion. This is a low specificity recognition, which generates low-affinity antibodies that easily end up activating autoimmune mechanisms. **(E)** Dysregulation of checkpoints: alteration of the CTLA4 and PD-1 autoreactivity checkpoints in T cells, resulting in the loss of physiological modulation or regulation of the autoimmune response.

The overproduction of autoantigens may well occur because the microorganisms produce enzymes that can break the extracellular matrix (i.e. fibrinogen, fibronectin, and type I collagen), creating “remnant epitopes” that act as autoantigens or because of an enzymatic modification of antigens (e.g. citrullination) that ends in the generation of autoantibodies (40). In health, the matrix metalloproteinases (MMPs) produced by the host have a role in tissue development, homeostasis and remodeling, cell migration and tissue healing, activation of immune cells and defense against pathogens, in a highly regulated form. When regulation is lost, high levels of these proteases lead to cellular destruction described in diseases of various kinds, from periodontitis to cancer (41), and why not autoimmunity (**Figure 2A**).

Microorganisms also produce MMPs causing direct tissue damage (close to 1%), but more importantly they activate circulating immune cells to produce MMPs which are responsible for the greatest amount of tissue rupture (42); then, chronic inflammatory processes lead to the production of cytokines and chemokines secreted by phagocytes, that recruit and stimulate leukocytes to release proteinases that act together with the MMPs (43). These events result in the proteolysis of matrix molecules and degradation of intact proteins into remnant fragments (2). These Remnant Epitopes Generated Autoimmunity (REGA model) explain the generation of autoantigens and their interaction with the T-cell receptor complex. In this model, cytokine and chemokine regulated proteases play a central role in the generation of autoantigens (44). Initially, evidence was documented for this model in multiple sclerosis (MS), however, it has been applied to other autoimmune diseases like rheumatoid arthritis (RA) and type I diabetes (45).

Recently the model was revised and reaffirms that it is an example of how remnant epitopes generate, maintain and regulate autoimmunity, in which the cells of innate immunity and not lymphocytes or their molecules initiate autoimmune reactions by cytokine-mediated proteolysis which consequently leaves remnant epitopes (46). Although REGA is not fully described in periodontitis, the high degree of destruction observed in periodontal tissues mediated by different proteinases makes it quite plausible.

On the other hand, the post-translational modification of proteins by the enzymatic deamination of arginine residues converts positively charged peptidyl arginine to neutral peptidyl citrulline (47). This process is called citrullination and is catalyzed by the enzyme peptidylarginine deiminase (PAD) (48). Various protein candidates for citrullination, such as keratin, fibrinogen, vimentin, fibronectin, and α-enolase have been identified (49).

Apart from being involved in many physiological processes (skin keratinization, brain development and in gene regulation *via* chromatin remodeling), citrullination can occur under pathologic inflammatory conditions associated with apoptosis, necrosis of neutrophils and NETosis (50). During the latter process, hypercitrullination of proteins is needed for the formation of neutrophil extracellular traps (NETs) (47) which are extracellular fibers generated by activated polymorphonuclear neutrophils, composed of nuclear constituents that extracellularly immobilize, disarm and kill microbial pathogens (48), meaning that under normal conditions NETs have antimicrobial function. However, uncontrolled NETs formation might contribute to tissue damage and provide a source of autoantigens; the citrullination of proteins and peptides has been linked to RA, primary open-angle glaucoma, nephropathy, MS, Alzheimer’s disease, and psoriasis (47).

The breakdown of immune tolerance in genetically susceptible individuals initiates the generation of anti-citrullinated protein antibodies in the synovia and contributes to the subsequent development of RA (49). These autoantibodies are known as ACPA and occur in ~ 70% of patients with RA. ACPA are known to be a sensitive and specific marker that can be detected in the circulation years before the clinical onset of the disease (47). During the clinical course of RA they strongly correlate with disease severity (50).

Five different PAD have been characterized in humans, each one with a different tissue distribution (50). *P. gingivalis* is the only known periodontal pathogen to produce PAD (40). The unique ability of *P. gingivalis* to produce PAD provides an association link between periodontal infection and RA (50). In a recent systematic review evaluating serum antibody levels against *P. gingivalis* in patients with or without RA, it is indicated that RA is often accompanied by the presence of an immune response against *P. gingivalis* (51). Thus, the formation of citrullinated proteins in the periodontium in a similar way to those formed in RA, may lead to think that the citrullination induced by periodontitis may play a role in the etiology of RA (52). Additionally, the finding of extra-articular citrullination and the production of ACPAS in periodontal tissues has been repeatedly reported. The presence of mRNAs for PAD-2 and PAD-4 has been found in gingival tissues in the presence and absence of inflammation, while anti-cyclic citrullinated protein antibodies are more frequent in the crevicular fluid of patients with periodontitis. Citrullination processes in the periodontal stroma is dependent on inflammation whereas citrullination in the gingival epithelium has been described as a physiological process (52–54) (**Figure 1A**).

#### Microbial Translocation

Body circulation is a closed system, actually, blood in healthy organisms was thought to be a “sterile” microenvironment (e.g. in the sense of absence of cultivable microorganisms) and bacteremia could be potentially life-threatening. However, the presence of a blood microbiome has been associated with diseases classified as non-infectious and it has been described that a large number of bacteria could use some alternatives as dissemination mechanisms including intracellular persistence (*Listeria monocytogenes, Salmonella typhimurium*, and *Yersinia pestis*), or using circulating neutrophils as Trojan horses (*Staphylococcus aureus*) (26).

Bacteremia leading to microbial translocation is a viable mechanism in all those systems in contact with the external environment, such as upper and lower respiratory tract, skin, digestive tract, and the oral cavity, from where not only the microorganisms can pass into the blood but also reach the intestine and induce multiple immune responses. Increased intestinal permeability, from where microorganisms or their metabolites could also reach the circulation, has been confirmed as a crucial mechanism in multiple autoimmune diseases (55) (**Figure 2B**).

RA is one of the clearest examples to explain bacterial translocation through different routes associated with alterations in the microbiome (both oral and intestinal) and the occurrence of autoimmunity (56) The intestinal microbiome has been proposed as an indispensable factor in the progression of RA (57). Citrullinated peptides have even been found in the colon, the lungs and synovial fluid of rheumatoid arthritis’ patients, supporting the idea that the mucosa of the colon can be one of the breaking sites of immune tolerance to citrullinated proteins (58). The role of the microbiota in the appearance or exacerbation of arthritis has been demonstrated in murine models (59) in which by inducing alterations in the intestinal microflora, production of proinflammatory cytokine-producing cells can be assessed at the intestinal and extraintestinal levels (60). From there they migrate to peripheral lymphoid tissues activating inflammatory processes that in turn result in systemic differentiation of B cells and the production of antibodies, which could trigger the disease *via* recognition of molecular patterns of the intestinal microflora (61).

Beyond RA and diabetes, increased intestinal permeability has been confirmed in inflammatory bowel disease (62) and other autoimmune diseases such as MS. *Helicobacter pylori* (a gastric microorganism) was found in kidney biopsies of patients with lupus nephritis (63), and antibodies against it react with extra-gastric tissues as glomerular capillary walls, ductal, renal tubular cells and glomerular basement membrane (64) for what they have been involved in the pathogenesis of different kinds of nephropathies considered immune complexes mediated diseases. This shows that in some way there is translocation of microorganisms, their components, their metabolites or immune products against them to local tissues, supporting their participation in the occurrence of autoimmunity, although the exact mechanisms of this relationship must be identified.

A recent experiment in which *P. gingivalis* was administered orally to C57BL/6 mice showed changes in the intestinal microflora, the barrier function and the immune system, which is reflected in an increased risk of systemic diseases characterized by, among others, low-magnitude systemic inflammation (65). Translocation is part of these mechanisms since bacterial periodontopathic deoxyribonucleic acid (DNA) has been found in various organs and tissues (66) as well as an increase in interleukin 6 (IL-6) in the bloodstream (67). The oral-intestinal connection has been also proposed as one more hypothesis to establish the link between periodontitis and systemic disease. In animal models, it has been shown that oral administration of *P. gingivalis* can induce intestinal dysbiosis (68) and result in endotoxemia and inflammation of the liver and adipose tissue, as well as an increase in the severity of collagen-induced arthritis mediated by an increase in T helper 17 cells (Th17) function at the intestinal level (65) (**Figure 1B**).

It has also been described in animal models of type 1 diabetes, that oral administration of *P. gingivalis* alters the intestinal microflora, inducing dysbiosis manifested in increased genera *Brevibacterium*, *Corynebacterium*, and *Facklamia*, aggravating glycemic control. In addition to the presence of microorganisms in the feces, an alteration of intestinal permeability was observed (due to action on tight intercellular junctions), as well as a dysregulation of the inflammatory response and glucose-fatty acid metabolism in the ileum and the liver (69). Thus, the translocation of oral microorganisms (including periodontopathogens) (69) and their products including citrullinated peptides in the case of *P. gingivalis* (48) is a recurrently cited mechanism in the interrelation between periodontal diseases, autoimmunity and other inflammatory diseases.

#### Molecular Mimicry

Molecular mimicry occurs when a self-antigen is so similar to an antigen from a microorganisms or other source in the environment that antibodies raised against that epitope will also bind to the autoantigen. It has long been clear that pathogenic microorganisms can potentially provide ligands that activate cross-reactivity with host-specific antigens (70, 71), which today is established as one of the most accurate links with the generation of autoimmune processes mediated by infections (72), but has also being recognized for a long time as a mechanism of evasion of microorganisms to the direct immune response (73, 74) (**Figure 2C**).

For homologous or even identical peptides to activate that T cell response, they must be available to be presented by antigen presenting cells (APCs) and must be properly processed by proteases and the machinery that performs this function. It is not unthinkable that the reduction of microbial diversity associated with inflammation and autoimmune diseases (75, 76) creates conditions under which microorganisms or their products can, with greater efficiency, traverse the epithelial barrier and be seen by APCs. Strong evidence is needed to demonstrate the role of commensals as antigen providers for these cross-reactions, to determine whether or not the role of molecular mimicry by commensal bacteria is related to the occurrence of autoimmunity (77).

There are multiple examples that prove the existence of molecular mimicry in the generation of autoimmunity related to different microorganisms, like: heart disease related to a chlamydia infection (78), MS due to infection with a virus in early stages of life that shares antigenic structures with tissues of the central nervous system such as myelin basic protein, inducing demyelination (79); patients with type 1 diabetes mellitus due to the homology in the sequence between the enzyme glutamate decarboxylase (GAD65) of the β cells of the pancreas with the enzyme P2-C related to the replication of the coxsackie B virus (80); rheumatic fever due to cross reaction between bacterial molecules: protein M (higher virulence factor of group A for *Streptococci*) and N-acetylglucosamine with myosin present in the myocardium (81); autoimmune uveitis (82), ankylosing spondylitis (83), Sjogren’s syndrome (84), systemic lupus erythematosus (SLE) (40), etc.

The oral cavity, especially in the presence of periodontal diseases, has proven to be a source of antigens both locally and systemically and molecular mimicry is undoubtedly one of the mechanisms by which it participates as a chronic infection in local and remote autoimmunity. The heat shock proteins (HSP) belong to a family of proteins conserved in prokaryotic and eukaryotic organisms during the evolutionary process, whose homology gives them a strong immunogenicity. In a chronic infection the immune response generated by human HSP60 and its bacterial equivalent, activates human vascular endothelial cells for the expression of E-selectin, intercellular adhesion molecule-1, and the vascular cell adhesion molecule-1. This generates the activation of endothelial cells, smooth muscle cells, and monocytes/macrophages for the production of IL-6 and tumor necrosis factor-α (TNF-α) (85).

High levels of human HSP can have a “toxic” effect on the immune system by stimulating pathological forms of activation as in the case of chlamydia infections and this has been defined as “immunovirulence.” The periodontopathogenic bacteria of the red complex have HSP homologous to GroEL from *Escherichia coli*. In other bacteria apart from them, such as *Agregatibacter acinomycetemcomitans*, the production of these HSP have been reported. Two subfamilies of proteins, GroEL and Dnak, are present in these bacteria in different amounts and molecular weights (86). The GroEL homologs are known as key molecules for autoimmune type reactions because of their similarity in sequence with human proteins, specifically with the human HSP60 protein present not only in periodontal cells but also in endothelial and muscle (87).

Multiple peptides from GroEL show more than 60% identity with human HSP60 (88) (**Figure 1C**). Antibodies anti-*P. gingivalis* GroEL HSP60 and to human HSP60 (indicating cross-reactivity) are detected in all samples of gingival tissue extracts from periodontitis and periodontally healthy subjects, but a higher frequency of seropositivity and a stronger reactivity is found in the periodontitis patients (64). Also, the existence of accumulated HSP60-specific T cells has been demonstrated in the gingival lesions of patients with periodontitis, with a strong proliferative T-cell response to human HSP60 in periodontitis patients compared with periodontally healthy control subjects. On the other hand, the proliferative response to *P. gingivalis* GroEL was much lower than that to human HSP60, which may indicate that *P. gingivalis* GroEL stimulates regulatory T cells (Tr cells), which play a critical role in the generation and maintenance of tolerance (89). The fact that even healthy patients were seropositive could indicate that these antibodies could act as a beneficial protective immunity to microbial HSP60 acquired by infection.

IgA-HSP60 levels are lower in periodontitis patients, but immunoglobulin G (IgG) class antibodies have been linked to antibody levels for *A. actinomycetemcomitans* and *P. gingivalis* (90). On the other hand, the levels of antibodies against human HSP60 and *P. gingivalis* GroEL in patients with atherosclerosis are significantly higher due to the presence of cross-immunological reactions, which would possibly facilitate endothelial pathology. This indicates that anti-*P. gingivalis* GroEL antibody can react against HSP60 expressed on injured endothelial cells. Antibodies elevated against HSP60 due to periodontal disease, may eventually become a risk factor for developing atherosclerosis in susceptible patients (91). Then, at the systemic level, the possibility of relating coronary and cerebrovascular diseases associated with this autoimmune mechanism has also been described, since elevated serum levels of antibodies against human HSP60, during the course of periodontal disease, may have a cross-reaction with gingival tissue, vascular endothelium and smooth muscle or the possibility of deteriorating pre-existing lesions (92) (**Figure 1C**).

Among other examples of molecular mimicry is the I-β2 glycoprotein which is important in the suppression of coagulation; some antibodies against the protein fraction TLRVYK of this molecule are related to thrombotic episodes. Leukotoxin C of *A. actinomycetemcomitans* has protein sequences (SIRVYK) homologous to those of glycoprotein I-β2. In patients with periodontal disease positive for *A. actinomycetemcomitans*, an antibody response was evidenced for the protein fractions SIRVYK of *A. actinomycetemcomitans* and TLRVYK of glycoprotein I-β2, being relatively equivalent values ​​and these two markers, in turn, higher in patients with periodontal disease regarding healthy patients (93). Under this concept, it was hypothesized that a chronic *A. actinomycetemcomitans* infection can increase thrombogenic antibodies against the I-β2 glycoprotein antibody by molecular mimicry, especially the IgG2 anti-SYRVYK response (94). In addition to the above, *P. gingivalis* also has a protein sequence (TLRIYT), as well as *T. denticola* (TLALYK), that have high homology to the I-β2 glycoprotein peptide, which may be another mechanism of molecular mimicry (95) (**Figure 1C**).

It has also been observed that anti-cardiolipin antibodies (aCL) from patients with periodontitis can be proinflammatory, promoting the activation of cell TLR4 pathways. These antibodies appear to be produced by molecular mimicry given their similarity to antigens of oral bacteria *P. gingivalis*, *A. actinomycetemcomitans*, and *T. denticola*, since all 3 bacteria have antigens with peptide sequences with significant homology to a cryptic binding site. Antibodies in serum to β2GPI protein, the target antigen of aCL, could indicate that circulating aCLs can induce or influence inflammatory responses at sites distant from the oral cavity (96).

Gingipains from *P. gingivalis* have been reported to be recognized by natural IgM and by being molecularly identical to the epitopes of anti-oxidized LDL (oxLDL) in malondialdehyde-modified LDL. Therefore, it may be that periodontal pathogens stimulate atherogenesis by activating autoimmune responses due to similar antigenic structures in the host, such as HSP or OxLDL, by molecular mimicry (97). After an immunological analysis to show the systemic levels of antibodies against *A. actinomycetemcomitans*, *P. gingivalis*, HSP 60, 65 and 70 and the OxLDL in plasma, it was found that the levels were higher in patients with periodontitis, however none of these markers of molecular mimicry decreased after periodontal treatment, even six months later. Thus, it can be thought that the increased risk of cardiovascular disease in periodontitis patients is associated with a complex and persistent immune response that may become resistant to periodontal therapy (98).

There are other related HSPs. Anti-HSP65 antibody levels have been reported in carotid atherosclerosis and coronary heart disease. Hypertension has also been associated with B cell activation and autoantibody production (anti-Hsp70, anti-Hsp65, anti-Hsp60, anti-AT1R, anti-α1AR, and anti-β1AR) (99).

Thus, molecular mimicry goes beyond the similarity of structures between the host proteome and the microbiome and includes genetic, environmental factors and alterations that are related to the selection processes of T cells in the thymus that allow the filtration of self-reactive cells (100), which makes this mechanism an interesting therapeutic target that is already being explored (101).

#### Superantigens

Microbial superantigens (SAg) induce the activation and expansion of a large number of T and B cells, which can result in the production of great amounts of regulatory and effector cytokines, modifying the host’s immune functions (102). They are mainly secreted by gram-positive bacteria and are capable of inducing pathological symptoms (103). This SAg binding mechanism activates a large proportion of T cells (up to 25% of all T cells) in comparison to conventional antigens (0.0001% of all T cells) (6, 7). SAg differs from regular antigens because interacts with the variable region of TCR-Vb chain and CMH-II in the antigen-presenting cell in a segment outside the antigen-binding site and induce the cell activation by cross linking of receptors (104). Therefore, SAg recognition by T cells has a significantly lower degree of specificity, as compared with their interaction with conventional antigens (105) (**Figure 2D**).

Findings from the late 1990s showed that T cells in periodontitis have a limited repertoire of expression of T cell receptor (TCR) variable region (V) gene products compared to those found in autologous blood which suggested that gingival T cells are not randomly mobilized from peripheral blood and that local events influence the T cell recruitment and expansion repertoire (106). In the gingival tissue of patients with periodontitis but not in healthy tissue, about 50% of all T cells express one or a few families of TCR Vb, leading to the hypothesis that the expansion of T cells in inflamed gums may occur through superantigens present in periodontopathic bacteria (107) (**Figure 1D**).

However, the results seeking to confirm the role of superantigens in associated bacteria and periodontal disease in the inflammatory response were controversial. Mathur’s study in 1995 showed that co-culture of peripheral blood mononuclear cells with *Prevotella intermedia* induces expansion of peripheral blood T cells expressing Vab2, Vb5, and Vb6, in patients with periodontitis and healthy individuals, a result that was not found for *A. actinomycetemcomitans* and *P. gingivalis* (108). These findings were confirmed by the expansion of Vb8, Vb12, and Vb17 cells in response to a strain of *Prevotella intermedia* (109). On the contrary, a study carried out in a murine model in which the proliferation of T cells in response to extracts of *A. actinomycetemcomitans, P. gingivalis, Prevotella intermedia* and *Prevotella nigrescens* showed that the extracts of these bacteria are not capable of activating T cells by means of superantigens (110). The role of T cell activation by superantigens has not been demonstrated for *P. gingivalis* that induces expansion of CD4 and CD8 Vb5.2 T cell clones but this activation appears to be antigen specific rather than by superantigen activity (111). In 2005 a review on the aspects of adaptive host response in periodontitis emphasizes that even though autoimmune reactions are evident in periodontitis lesions, the role of superantigens in periodontitis is unclear (112).

Even when in periodontitis the hypothesis of activation by superantigens and their ability to alter the response could not be demonstrated and was abandoned, the mechanism is still relevant to explain the pathogenesis of diseases where the host microbiome interaction is crucial, such as the autoimmune sequelae of streptococcal diseases, toxic shock syndrome with its characteristic dysregulated cytokine storm, allergic inflammation, and tonsillar hyperplasia, among others where bacterial infections can mimic autoimmunity (113–116).

#### Activation and Inhibition of Receptors Related to Autoimmunity

Overregulation of inhibitory receptors (IR) such as CTL4 (cytotoxic T lymphocyte–associated protein 4) and PD-1 (programmed cell death protein 1) represents an essential mechanism by which immune responses are controlled to maintain immune homeostasis and prevent autoimmunity. The involvement of these checkpoint mechanisms has also been described in evading anti-tumor immunity, they are recognized as one of the signs of cancer (117) and its blockers are widely studied in antitumor therapy (118). Chronic infections, also induce a variety of immunoregulatory mechanisms such as the production of anti-inflammatory cytokines, activation of regulatory T cells (Treg), and the expression of these checkpoint immune molecules (119).

CTLA4 is expressed on T cells and interacts with CD80/CD86 on APCs limiting T activation and leading to anergy. PD-1 is also expressed in T cells and interacts with PD-L1 and PD-L2 expressed in APCs and tumors by sending a negative message to the T cell that leads it to T cell-exhaustion. Check point inhibitors have been shown to increase ex-vivo effector T cell responses on patients with bacterial, viral, and parasitic infections, including human immunodeficiency virus, tuberculosis, and malaria, making their study an open door for the development of therapeutics for infection control (120). Check points bind immunity regulatory mechanisms such as Tregs, myeloid derived suppressor cells (MDSCs), M2 macrophages, regulatory cytokines such as IL-10 and transforming growth factor beta (TGF-β) to prevent tissue damage, thus their failure is related to infectious and inflammatory diseases (121) (**Figure 2E**).

Humans with CTLA4 mutations develop widespread immune dysregulation. Antibodies that block checkpoints, also bring adverse effects such as the breakdown of self-tolerance, which highlights its role in the physiological modulation of immune responses and therefore has been used as a target of agonist agents to restore tolerance in the context of autoimmunity (122). In periodontitis, an increase in the expression of CTLA4 on CD4^+^ T-cells has been found related to the presence of periodontitis and, more recently, the study of these IR has specifically focused on the context of the relationship of possible polymorphisms of the gene that encodes it with susceptibility to specific forms of periodontitis in different populations (123–125) (**Figure 1E**).

Polymorphisms in PD-1 have been linked to susceptibility to autoimmune diseases including SLE, atopy, RA, and progression of MS (126). PD-1 expression in periodontitis has been evaluated to explain the presence of immunosuppression (127) and to be used as a therapeutic target (128). No evidence of their analysis was found in periodontitis in relation to autoimmunity, but the results of the studies open an interesting window. In an ex-vivo murine model, the expression of PD-1/PD-L1 in primed T cells with *P. gingivalis* showed an increase in PD-1 expression in CD4^+^ T-cells in the presence of *P. gingivalis* activation when compared to controls, but these differences were not found in CD8^+^ T-cells. This overexpression was higher in competent IL-10 cells (capable of producing IL-10), suggesting increased suppressive activity of the interferon gamma (IFN-γ) response in these cells. The study concluded that in addition to IL-10 production, up-regulation of the PD-L1/PD-1 inhibitory signal may be another mechanism used by *P. gingivalis* to suppress CD4^+^ T-cell response, possibly contributing to the persistence of the bacteria in the body (129) (**Figure 1E**).

### Mechanism of Autoimmunity Dependent on The Host Response

#### Bystander Activation

An adaptation mechanism of innate immunity has been described as bystander activation. This occurs as a consequence of infected cells which alert and give instructions to neighboring uninfected cells to produce inflammatory mediators either by direct cell to cell contact (mediated by GAP junctions by modulation of connexin) or by paracrine action (soluble signals). This mechanism may allow the immune system to overcome the pathogen’s ability to disarm the immune signaling pathways in infected cells. The mechanism has been described in viral and bacterial infections. In addition to communication through microorganisms, cytokines or pathogen-associated molecular patterns, macrophages have been described *in vitro* as releasing active inflammasomes to establish contact (130).

Recent evidence could indicate that there is an underestimation of the role of innate pathogen-associated receptors in T cells (131), since they have been shown to have non-classical activation patterns. That goes beyond the exclusive reliance on antigenic recognition through TCR. Both the γδ T lymphocytes and the mucosal associated invariant T (MAIT) cells, and the conventional αβ CD4^+^ and CD8^+^ T-cells in mice and humans express TLRs, demonstrating that the cells of adaptive immunity also use these innate signaling pathways leading to the promotion of T helper cell-dependent inflammation through TLRs (132); this makes us think of these receptors as important regulators of the disease during infection.

This process of activation independent of TCR antigen presentation, also occurs by bystander activation, and was described more than 2 decades ago (133) based on the T cell expansion observed in viral infections. It is a heterologous activation of specific non-antigenic lymphocytes, mediated by indirect signals that favors the inflammatory microenvironment such as costimulatory receptor ligands, cytokines, chemokines, pathogen-associated molecular patterns, extracellular vesicles and microbial particles. Bystander activation occurs in both T cells and B cells, in which the stimulation is independent of the BCR. Cells infected by bacteria or viruses can induce activation of uninfected cells through soluble signals such as cytokines, co-receptor expression (example TLR, CD122, and NKG2D), and intercellular communication mediated by GAP junctions (100).

**Figure 3:**
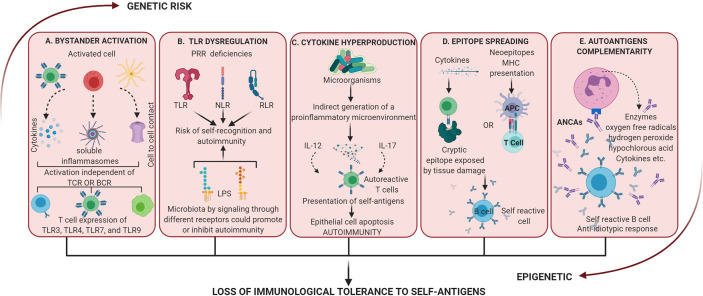
Dysregulation of the activation of inflammatory processes mediated by a dysbiotic microbiota. These mechanisms alone or together lead to the loss of immunological tolerance to self-antigens. **(A)** Bystander activation: an “infected” cell can activate neighboring uninfected cells by cytokine release, the generation of soluble inflammasomes by macrophages, or by cell-cell contact *via* GAP junctions. Bystander activation can induce non-classical activation patterns such as T helper cell-dependent inflammation through TLRs. **(B)** Dysregulation of TLR: these receptors have evolved to sense nucleic acids which comes with the risk of self-recognition and autoimmunity. At the same time different LPS could promote or inhibit autoimmunity by signaling through different receptors. **(C)** Cytokine hyperproduction: proinflammation activated by commensal microorganisms can increase the response of T cells to self-antigen. Also, the differentiation of self-reactive Th17 cells has been related to apoptosis of intestinal epithelial cells induced by microbial stimulation. **(D)** Epitope spreading: it is related to autoimmunity by 2 mechanisms, first inflammation allows T cells to recognize cryptic epitopes and activate B cell complementarity (independent of the antigenic presentation) and second antigen processing and presentation to activate T cell which reciprocally activate B cell (no tissue damage present). **(E)** Autoantigens complementarity: the activation of the autoimmune response does not occur by the autoantigen but its complementary protein-peptide initiating the production of antibodies which activate an anti-antibody response (anti-idiotypic response). ANCAs is an example of autoantibodies generated by these mechanisms and activation of PMNn produces enzymatic and proinflammatory products that result in dysregulation and tissue damage.

Lipopolysaccharides (LPS) can activate T lymphocytes non-antigen specific. Furthermore, they can activate dendritic cells by up-regulation of CD86 and IFN-γ production. Innate immunity mediators such as DC and natural killer cells (NK), induce bystander activation of T cells in response to TLRs agonists through the production of IFN-α/β/γ (134) (**Figure 3A**).

Multiple autoimmune diseases are associated with bystander activation: RA, SLE, type 1 diabetes, MS, autoimmune hepatitis, and autoimmune thyroid disease, among others (100). Specifically, for direct activation of T cell signaling through TLRs, there is evidence indicating that recognition of T cells by TLR3, TLR4, TLR7, and TLR9 is related to disease aggravation in models of infection, cancer, and autoimmunity (135).

All the mechanisms described above converge in the bystander activation mechanisms of T cells, since it involves the participation not only of innate receptors but of other factors such as superantigens by polyclonal activation of T cells, which in turn triggers a storm of cytokines. Additionally, molecular mimicry, dual TCR signaling, and virtual memory T cells (136) (which has been observed in experimental animals) are other none classical T cell activation pathways (135). Thus, this mechanism, although without a demonstrated specific involvement in periodontal disease, hypothetically could participate in the pathogenesis of periodontitis from the activation of host responses to dysbiotic changes, which involve innate immunity, as in the case of dysregulation of TLRs and all the branches of immunity as in the hyperproduction of cytokines (**Figure 1F**).

#### Dysregulation of TLRs

Deficiencies in pattern recognition receptor (PRR) signaling that controls homeostasis at the level of composition of the microflora, lead to increased susceptibility to certain diseases (24). When there is inappropriate activation of nucleic acid-sensing, TLRs can cause pathogenic inflammation and autoimmunity (137, 138). TLRs that have evolved to sense nucleic acids and the recognition of microbial DNA or RNA, clearly represents a key strategy by which the innate immune system detects infection; however, the detection of nucleic acids comes with the risk of self-recognition and autoimmunity (139).

For TLR4, a contribution has been suggested in the improvement of autoimmune diabetes in NOD mice, since the deletion of signaling genes as well as the knockout of TLR4 itself have been seen to cause an increase in the incidence of autoimmune diabetes in these animals (140). On the other hand, the deletion of the TLR2 gene reduces the incidence of autoimmune diabetes, suggesting that differences in LPS derived from the microflora between individuals, may prevent the education of their immune tolerance, which would trigger autoimmunity (141). Therefore, the microbiota could promote (through TLR2) or inhibit (through TLR4) the autoimmune responses signaling by different receptors (22) (**Figure 3B**).

The activation of oral epithelial cells, cells of the innate and acquired immune response through CD14, TLR2, and TLR4, are considered a relevant event in the generation of the inflammatory response by the production of proinflammatory cytokines by binding with LPS of periodontopathic microorganisms in periodontal disease. The altered expression of these receptors has been reported in periodontitis (142, 143). TLR 1, 2, 3, 4, 5, 6, and 9 are differentially expressed in connective tissue and epithelium in periodontitis and in greater numbers in periodontitis patients compared to healthy tissue (143) (**Figure 1G**).

Despite these findings, a direct role of TLRs activation as inducers of periodontal damage has not been consistently demonstrated. Just as TLRs expression is found in the inflamed tissue, negative regulatory mechanisms have also been found including the production of soluble forms of these receptors, which inhibit signaling pathways by blocking their ligands (144) (**Figure 1G**). Soluble CD14 levels were significantly higher in gingivitis and periodontitis compared to oral health (145). TLR2 soluble in saliva shows an inverse correlation and TLR4 soluble directly with clinical parameters in periodontitis (146). Additionally, tolerance to the action of endotoxins has been demonstrated by repeated stimulation with periodontopathic bacteria (147). LPS-stimulated cells of the human periodontal ligament have decrease expression of TLR2, TLR4, IL-6, and IL-8 (148).

It has recently been described in a mouse model of periodontitis with RA, that the modulation of cathepsin K-mediated TLR9-related autophagy, can decrease bone destruction in periodontitis promoted by RA, by modulating the infiltration of macrophages, TLR9, autophagy proteins (TFEB and LC3) and inflammatory cytokines (149) (**Figure 1G**).

Collectively these findings show that TLRs in the periodontium and their regulatory mechanisms can be activated or inhibited by specific ligands derived from periodontal bacteria (150). Therefore, a specific role between bacteria or its genetic material and the innate host response by TLRs that leads to the occurrence of autoimmune responses has not been established but cannot be ruled out.

#### Amplification of Immunity by Hyperproduction of Cytokines

The amplification of immunity by hyperproduction of cytokines was related to the appearance of autoimmune responses after discovering that the production of proinflammatory cytokines activated by commensal microorganisms (specifically IL-12 at the level of gut-associated lymphoid tissues) can increase the response of T cells to self-antigens (22). IL-12 is attributed a dual role in periodontal disease; in gingival fluid (151, 152), gingival tissue and serum (153), it has been associated with the severity of periodontal destruction. Additionally, the polymorphism of the IL-12 gene is associated with susceptibility to the development of chronic periodontitis (154) and studies in IL-12 deficient mice with *P. gingivalis*-induced periodontitis show less bone resorption which supports the participation of this cytokine in the development of bone destruction in the disease (155). On the other hand, IL-12 induces the expression of immunosuppressive molecules, such as human leucocyte antigen (156) and indoleamine-pyrrole 2,3-dioxygenase (157) through an IFN-γ–dependent pathway. IL-12-induced immunomodulation may be an important mechanism that helps regulate the host’s immune response and maintain tissue homeostasis during periodontal inflammation. These findings suggest positive and negative influence of IL-12 on periodontal disease (158). However, it is not known how dysbiosis of the periodontal microbiota can guide IL-12 function towards a protective role or proinflammatory function. Understanding the role of the microbiota in this mechanism could be used to regulate or control the evolution of periodontal disease.

Despite the regulatory processes in which cytokines participate, microorganisms are generally related to autoimmune responses by indirect generation of a proinflammatory microenvironment where this expression of cytokines would favor the presentation of self-antigens leading to autoimmunity (17). Apoptosis of intestinal epithelial cells in response to microbial stimulation has been shown to increase the presentation of self-antigens resulting in differentiation of self-reactive Th17 cells (159). Although this mechanism has not been specifically described for periodontopathogens, the tropism of the pathogen could determine the specific location of the inflammatory disease (**Figure 3C**).

It has been studied in various germ-free animal models how the production of cytokines by cells of the innate response such as DC, macrophages, neutrophils and NK after their activation by microorganisms, are necessary for the development of autoimmunity. For example, macrophages isolated from germ-free mice release lower levels of TNF-α and higher levels of IL-10 when stimulated with bacterial LPS (160). NK function is also altered under these conditions, possibly due to the inability of monocyte-derived cells to produce type I interferons in response to microbial stimuli (77). Additionally, the decrease in the number of Th17 cells in the intestine in germ-free mice improves autoimmune glomerulonephritis (22).

IL-17 is of particular interest in the pathogenesis of periodontitis because it is involved in both the inflammatory response and protective immunity against microorganisms (161, 162). Constant signaling through the IL-17 receptor can transform a protective acute inflammatory response into a chronic immunopathological response (163).

In periodontal disease, both IL-17 and IL-17F activate cells of innate immunity as neutrophils and cells from surrounding non-immune tissues (fibroblasts and epithelial cells), by activating the transcription factor NF-κB. IL-17 can activate both fibroblasts and endothelial cells to increase secretion of IL-6 in the presence of TNF-α (**Figure 1H**). IL-6 and RANTES are recognized factors for the progression of gingivitis to periodontitis. RANTES is produced by gingival fibroblasts after challenge with IL-1β and TNF-α, inducing recruitment of monocytes in the tissue; it is also a chemo-attraction factor of Th1 profile cells. The concentration of IL-6 and RANTES is high in deep (≥6 mm) periodontal pockets (164).

Although the mechanisms are not clear, there is evidence to suggest that *P. gingivalis* promotes an IL-17 rich environment, as a mechanism for obtaining nutrients that come from the degradation of oral tissues caused by inflammation (165). The Th17/Treg ratio in autoimmunity and Th17-mediated immunity is important for maintaining hematopoietic and mucosal homeostasis. Altered homeostasis between Th17 and Treg has been implicated in several autoimmune diseases, so the relationship between Th17 effector cells and Treg must remain in balance to preserve functional immunity and host health (166). Additionally, the trans-differentiation of Treg cells into cells that produce IFN-γ similar to Th1, and from Th17 cells that produce IL-17 to IFN-γ, has been implicated in the pathogenesis of several autoimmune diseases (167–169) (**Figure 1H**). Such trans-differentiation process has been demonstrated in a mouse model inoculated orally with *P. gingivalis* at 4-day intervals to simulate persistent dysbiosis. Results showed that in this environment of persistent dysbiosis as occurs in periodontitis, the CD4^+^ T lymphocyte-mediated immune response evolves from one initially dominated by IL-17A to one that is predominantly IFN-γ-producing, in a response generated *de novo* by Th1 cells. A small proportion of Treg cells expressing IL-17A on day 28 disappear on day 48. This evolution of dysbiosis and the inflammatory environment on day 48 by *P. gingivalis*, induces a transdifferentiation of Th17 and IFN-γ expression. The components of the microbial biofilm or host cells under the influence of such a microbial environment responsible for driving Treg or Th17 transdifferentiation in the oral environment are not known (170). Changes in microbial communities from the initiation of an accumulation and conformation of non-specific oral biofilm to subsequent dysbiotic changes, rather than a simple increase in microbial load, are necessary for the activation of disease-causing Th17 cells in mice and in humans (171). Therefore, transdifferentiation mechanisms may contribute to the transition from active inflammation to a resolution phase at sites with periodontitis.

#### Epitope Spreading

In many immune responses the initial antigens and epitopes of those antigens that are recognized by adaptive immunity are limited. Over time, the responses of T and B cells can grow to include many other epitopes and additional antigenic molecules, this process is known as epitope spreading. Under normal conditions, by expanding the antigenic epitopes recognized by the immune system, the response to these foreign antigens is optimized, allowing neutralization *via* antibodies, recognition by various immune cells, and clearance of the pathogen (172). The epitope spreading is essential for the development of normal adaptive immune responses, but in turn contributes to the immunopathological processes of infection-induced autoimmunity.

There are 2 mechanisms for the epitope spreading to occur in autoimmunity, one independent of the antigenic presentation, in which inflammation and cytokine activation are sufficient to allow T cells to recognize cryptic epitopes and activate B cell complementarity (173), and the other dependent on binding to an APCs that occurs when there is no tissue destruction, and relies on processing and presentation to activate T cell which in turn reciprocally activate B cell (174). Molecular mimicry and cross reactivity B cell epitope spreading after mimicry, is a mechanism by which the body initiates the breakdown of self-tolerance, leading to the development of autoimmune diseases. More is known about T cell spreading epitope than B cell epitope in disease (172). Therefore, it all begins with molecular mimicry against the dominant epitope, resulting in an auto-response directed against a neo-epitope. Intra-intermolecular epitope spreading is one of the four mechanisms on how antigens of infectious organisms may propagate into various human autoantigens to trigger adaptive autoimmune responses. Substantial evidence supports the hypothesis that tissue damage can lead to epitope spreading, which can then contribute to ongoing disease (175) (**Figure 3D**).

The peptide 19 from *P. gingivalis* HSP60 (Pep19) has been studied as one of the dominant peptides, from which, the epitope-specific immune response to subdominant epitopes could be sequentially diversified towards autoimmune responses directed against human neoepitope in periodontal disease induced by *P. gingivalis* and in autoimmunity (91). The mechanism as Pep19 directs the epitope spreading towards the formation of autoantigens in chronic periodontitis or in experimental periodontitis induced by *P. gingivalis* is not clear. Animal and human model research has described that in both young, systemically healthy humans and mice, there is a unique dominant immune response against Pep19, which persists in the presence of chronic periodontitis and autoimmunity, without subsequent replacement by response to subdominant epitopes (87, 174). Pep 19 generates sequential spreading epitope to Pep19 subdominant autoantigens of human HSP60 (Hu19) in healthy subjects and mice, to P9 autoantigens of human HSP60 (Hu9) and to oxidized low-density lipoprotein (ox-LDL) (neoantigen) in the Periodontitis induced by *P. gingivalis* as well as in autoimmunity. The proliferative activity of T cells to the autoantigens Hu19, Hu9 and ox-LDL, and the cross reactivity of Pep19 monoclonal antibodies to these epitopes are proposed as the responsible cellular and molecular mechanisms (175) (**Figure 1I**).

Pep19 has also been the object of study for immunotherapy as a target of Treg cell subsets in search of generating tolerance. Naive cells CD4^+^CD25^+^CD45RA^+^ Tregs and the epitope spreader Pep19 were proposed as cell and molecular targets for a scheme of antigen-specific Tregs-based vaccination in collagen-induced arthritis (177).

#### Autoantigens Complementarity

The theory of autoantigen complementarity (described in the 1990s) is based on the fact that the autoantigen is not the one that triggers the events that lead to autoimmunity, and that what activates it is its complementary protein-peptide (178). This initiates the production of antibodies which in turn activate an anti-antibody response or anti-idiotypic response. The anti-idiotypic antibody reacts with the autoantigen which has a sequence complementary to that of the initiator antigen. This mechanism was described from the study of cytoplasmic anti-neutrophil antibodies (ANCAs) directed against proteinase 3 (PR3) of neutrophil granules (179). It was discovered that patients not only had antibodies against PR3 but antibodies against peptides encoded by the noncoding band of genes encoding the PR3 autoantigen (178). This means that the initiator of the autoimmune response is not the autoantigen but rather the complementary protein in the microorganism that induces the idiotypic antibody response (**Figure 3E**).

ANCAs are defined as autoantibodies of the IgG type mainly directed against granular components of the neutrophil, specifically on proteolytic enzymes located within the azurophil or primary granules. Initially, their production was attributed to the polyclonal activation of autoreactive B lymphocytes but now they are considered as antibodies generated by autoantigens complementarity (180). When the binding between the ANCAs and target antigens occurs, an activation signal is emitted to the neutrophil for the release of enzymatic products (collagenases, elastases), oxygen free radicals, hydrogen peroxide, hypochlorous acid, hydroxyl radicals and nitrous oxide; cytokines and pro-inflammatory substances such as: IL-1β, TNF-α, platelet activation factor, thromboxane E2, and leukotrienes. This activates autocrine and paracrine responses to other cell types without regulation, added to the inactivation of enzymatic inhibitors, resulting in evident cell damage (181).

The apoptosis of neutrophils and their subsequent tissue removal is a vital function that must be fulfilled in order to not break the balance as apoptosis of neutrophils is essential for controlling the duration of early inflammatory response and thus limiting the local tissue damage that can result from prolonged activation of neutrophils. Defects in apoptosis or in the process of removal of apoptotic cells could lead to exposure of these cellular fragments to immune system and activating a humoral immune response (182). Thus, another mechanism by which the ANCAs exert their function is by preventing apoptosis and the removal of apoptotic neutrophils, generating a sustained immune response (183). This is a topic that has recently gained strength to explain the pathogenesis of autoimmune diseases characterized by a defect in apoptotic cell clearance and in the resolution of inflammation (i.e. granulomatosis with polyangiitis, an autoimmune necrotizing vasculitis associated with ANCAs), where PR3 that acts as an autoantigen impaired C1q enhancement of apoptotic cell uptake by altering the elimination of apoptotic bodies (184).

Initially described in systemic pathologies such as RA (185–188) and SLE (189–191), towards the end of the 1990s ANCAs were related to periodontal disease, due to the relationship established between these diseases and periodontitis. Novo et al. would be the pioneers in mentioning this mechanism in periodontal disease visualizing p-ANCA immunofluorescence patterns (191).

In the periodontal environment, ANCAs also seems to interfere directly with the apoptosis of neutrophils present in the lesion (192) (**Figure 1J**). When the oxidation of α-1-trypsin (inhibitor) occurs, this leaves free Proteinase 3 to be targeted by the enzyme attack. As a result, there will be high levels of substances and protein products mediating periodontal damage in the environment, such as: collagenase, gelatinase, cathepsin B and D, elastase, β-glucuronidase, myeloperoxidase, and lysozyme (**Figure 1J**). In addition, a greater number of molecules will be found, such as adhesins, selectins, and integrins that will allow greater adhesion to the vascular endothelium. Even so, it has been described that in early stages of periodontal disease when there is no polyclonal activation of B cells there is no presence of ANCAs (193).

In 2006, 15 years after the beginning of the study of ANCAs in periodontitis, a model was proposed to explain the pathogenesis of periodontal disease from the concept of ANCA associated autoimmunity. Based on multiple studies, the authors founded their model on the following points: 1. Genetic factors similar for periodontal diseases and other ANCA-associated diseases like Wegener’s granulomatosis, RA and, to a lesser extent, LES; 2. The activation of the cells bearing the ANCA antigens (neutrophils and monocytes) leading to an inflammatory response and eventually resulting in immense bystander damage; 3. The damage to other cells bearing the target antigens such as endothelial cells; 4. A loss of function by the direct inactivation of the antigen to which the ANCA bind; 5. The induction of the respiratory burst and the degranulation of neutrophils mediated by ANCA with the consequent release of enzymes and various metabolites capable of producing damage to the periodontal connective tissue; and finally 6. The local or systemic presence of superantigens that could ensue from periodontal pathogens and that may stimulate monocytes/macrophages to secrete proinflammatory cytokines and elevated levels of TNF-a reported in ANCA^+^ patients. The publication concludes that even though the evidence supporting the model puts periodontitis in the spectrum of diseases where autoimmunity mediates damage, further long-term prospective controlled studies were required to validate the ANCA-mediated periodontal destruction model; such studies were never done (183). Despite this type of approach, there is no direct evidence to support the participation of ANCAs in the tissue damage observed in periodontitis.

### Relevance of Auto-Antibodies in The Autoimmune Damage

**Figure 4:**
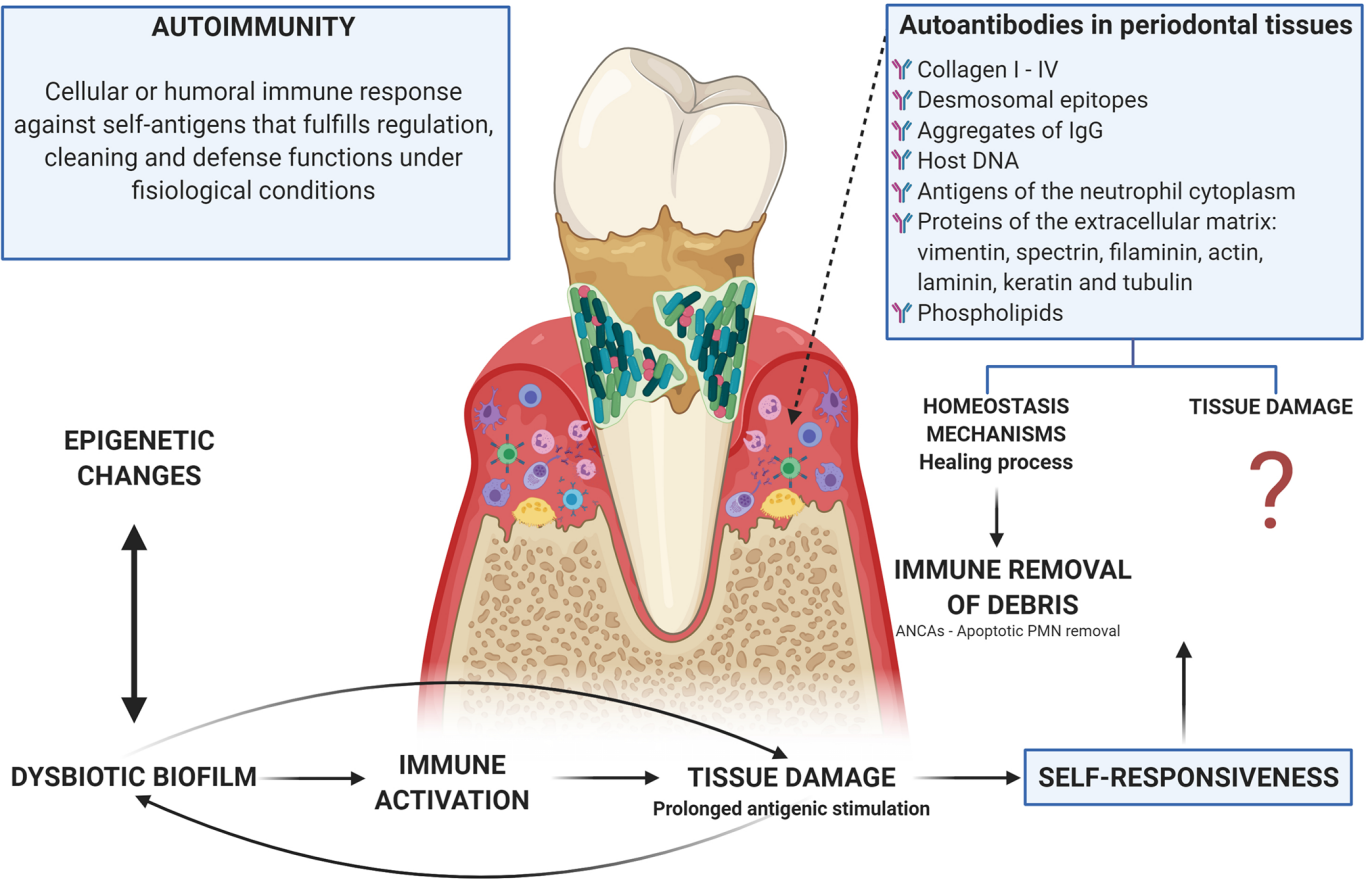
Autoimmunity in periodontitis: tissue damage related or homeostasis mechanisms? There is an evident relationship between the dysbiotic microbiota, the activation of inflammatory mechanisms, and the tissue damage observed in periodontitis, which leads to prolonged antigenic stimulation due to tissue breakdown with the release of matrix proteins. In turn, microorganisms use these products of tissue damage as a nutritional source. This persistence of both self and exogenous (microbial) antigens leads to the occurrence of autoimmunity, which can be understood as a mechanism by which debris generated by tissue damage are removed through the generation of autoantibodies (including ANCAs as a mechanism for the cleaning of apoptotic bodies after the activation of phagocytic cells) or as a damage machinery, although the evidence for the participation of autoimmune mechanisms in the tissue damage observed in periodontitis is poor and lacking analysis. Additionally, microorganisms and their products can alter the activity of epigenetic enzymes at the same time that these epigenetic changes can induce dysbiosis, thus epigenetics being a determining factor in the occurrence of autoimmunity due to a direct relationship with the breakdown of immunological homeostasis.

Autoimmune responses are part of multiple inflammatory processes including primary immunodeficiencies, in which the classic sign is the presence of autoantibodies. Autoreactive cells are found in healthy individuals and do not normally cause pathology, this could be due to low precursor frequency or regulatory controls that exceed them; but, in genetically susceptible individuals, regulation of activated autoreactive lymphocytes during tissue damage can be faulty and the pathogen can lead to disease *via* epitope spreading (175). Thus, 2 scenarios can arise: 1. An immune response to a persistent antigen with direct lysis that could cause damage to the own tissues (175); or 2. That healthy individuals having constitutive autoreactive cells indicates that they have a protective role. In fact, B cells produce antibodies that recognize their own epitopes, that have been described as beneficial for their participation in the cleaning of apoptotic bodies and the development and homeostasis of B cells (100) (**Figure 4**).

The study of self-responsiveness in the occurrence of tissue damage linked to the presence of microorganisms is complicated. It has been described that the participation of B-cells in the microbiome host interaction at the intestinal level, goes beyond the promotion of Breg cell differentiation and its production of IL-10 through the activation of IL-1β and IL-6; commensals seem to play a key role in the removal of autoreactive B-cells from the gut associated lymphoid tissue, a process that is defective in SLE and RA, so a lack of regulatory promoting bacteria that compromises synthesis or secretion of immunoglobulins could be hypothesized (40).

Amyloid fibers (curli fibers), a major extracellular matrix protein produced by bacteria and a component of bacterial biofilms, are capable of binding to extracellular DNA and forming curli/DNA complexes that lead to immune activation and the production of autoantibodies (40), establishing an important relationship of bacteria in biofilms with the possible immune dysregulation characteristic of autoimmunity responses.

In periodontal disease, antibodies have been found against almost all types of collagen (I to IV), desmosomal epitopes, aggregates of IgG, host DNA, antigens of the neutrophil cytoplasm, proteins of the extracellular matrix (vimentin, spectrin, filaminin, actin, laminin, keratin, and tubulin) and phospholipids (93, 191, 194, 195). It is possible that due to the prolonged antigenic stimulation caused by the rupture of the tissue and the release of the proteins of the extracellular matrix (a secondary event to the inflammatory process induced by microbial dysbiosis), these antibodies that can be considered as part of tissue homeostasis, suffer a change in isotype from IgM to IgG or IgA, while increasing concentration at the site of local inflammation (196, 197). However, IgG anti-collagen type I antibodies are present in gingival fluid during the inflammatory healing process (198), what is opposed to their participation in the damage and supports a possible role for instance in the regulation through what we could call immune removal of debris. Anti-desmosomal antibodies then appear to be a normal part of the immune repertoire (194) (**Figure 4**).

On the other hand, the levels of anti-desmosome antibodies are higher in sites with active periodontitis compared to unaffected sites in patients with periodontitis (194). Similarly, levels of anti-type I collagen antibodies are higher in gingival tissue than in peripheral blood (199), and IgG and IgA isotypes are found in higher concentrations in extracts of gingival tissue than in autologous serum while there are no differences in IgM levels (200). Anti-collagen antibody-producing B cells are found primarily in gingival tissue and in very low amounts in peripheral blood from patients with periodontitis (201). Additionally, serum levels of anti-CD24 IgG antibodies, a surface glycoprotein involved in cell-cell signaling and adhesion in epithelial tissue, have been reported to correlate with a more favorable clinical status in patients with periodontal disease (202).

Finally, one of the immune system functions, related to damage in autoimmunity by the formation of immune complexes, is the complement activation. The relationship between the complement system and autoimmunity appears paradoxical as both the deficiency and the activation, contribute to induce autoimmune diseases (203). The role of the complement system in enhancing infective diseases in secondary complement deficiencies, was mainly demonstrated in patients affected by autoimmune diseases. It has been suggested that the early part of the classical pathway, activated by the immune complex, plays a protective role against the development of SLE, whereas central and terminal component can contribute to disease development (204, 205). Recently, complement also emerged as a critical player in adaptive immunity *via* its ability to instruct both B and T cell responses, it has an impact on T cell responses that now permits to see this system functions also within cells and it´s involvement in regulating basic processes, predominantly those of metabolic nature (206). The presence of complement deposition in affected tissues, decreased levels of complement proteins (207) and high levels of complement activation fragments in the blood and vessels (208) have been documented related to autoimmunity.

Although complement activation has been described as an important mechanism in tissue damage in periodontal and peri-implant disease (209, 210), the most accepted interpretation of its activation is the capacity of the periodontopathogens that use it as a mechanism for tissue degradation in favor of the release of nutritional components, and thus the maintenance of biofilms (211, 212) and not directly as a mechanism of tissue damage.

### Epigenetics and Autoimmune Responses

Epigenetics refers to heritable genomic expression without alterations in the original DNA sequence (3) leading to remodeling of the chromatin and activation or inactivation of a gene (213). There have been described three epigenetics modifications: DNA methylation, histone modifications, mainly by short-chain fatty acids and microRNA (miRNA) regulations (3). These processes are potentially reversible and transient. It can be induced or altered by environmental factors that modulate and affect the gene expression and functions (213). Epigenetic regulations could break the immunological homeostasis and result in the development of autoimmune diseases (40) such as SLE, RA (214), Systemic sclerosis (215), Sjogren syndrome (216), etc, among others due to the inflammatory responses mediated by an increase in the number of iNKT, less reactivity of the NK, and alteration of production IFN type I, IL-6, IL-12, IL-18 and TNF-α (22).

SLE is the most studied autoimmune disease correlated with epigenetic modifications, a pattern of robust demethylation of interferon signature supporting a pathogenic role for neutrophils in lupus has been suggested, as well as a model whereby DNA from lupus neutrophils externalized by NETosis enhance type-I IFN production *via* TLR-9 stimulation by hypomethylated DNA (217). Other epigenetic modification described for the SLE are DNA methylation and histone modifications which regulate gene expression in mature T cells for different genes such as CD11a, perforin, CD70, and CD40LG are described in the literature (218), as well as overexpression of IL10 in T cells from SLE patients leading to an increase of specific autoantibody production and tissue damage (219).

Microbial metabolites can affect the activity of epigenetic enzymes or act as necessary substrates for epigenetic modifications. ncRNAs or epigenetic enzymes of the microbiome, can translocate to host cells influencing their gene expression. These epigenetic changes can also induce dysbiosis (40). This leads to the conclusion that the microflora is epigenetically regulated by the expression of genes that buffer autoimmune inflammation but increases the ability to respond to external challenges (22). All exposed responses could be microorganism-specific, depending on host susceptibility and lifestyle factors, as well as varying from animal to human models (**Figure 4**).

It has been established that both genetic and epigenetic factors play indispensable roles in the pathogenesis of periodontal disease (220, 221). Genetic background is necessary for disease onset, but it is insufficient for disease development. Epigenetic modifications also participate in the pathogenesis of periodontitis in genetically predisposed individuals (3) (**Figure 2**).

The post-translational modification of histone proteins in chromatin and the methylation of DNA are the two primary epigenetic mechanisms in periodontitis (3, 213). In addition, many environmental factors may have profound effects on the epigenetic changes and induces susceptibility to disease (3).

## Discussion

The recognition of the microbiota role in autoimmune responses has led to greater clarity in patterns of common mechanisms between autoimmunity and primary immunodeficiencies in humans (222). After consolidating the information, the question is: Are autoimmune responses in periodontitis a harmful machinery leading to tissue damage or the dysregulation of the immune mechanisms generated in a dysbiosis-induced inflammatory response, or normal physiological responses to the local presence of microorganisms?

To analyze the occurrence of the autoimmune mechanisms in periodontal disease, it is necessary to start from several facts at the level of periodontal tissues which establish the relationship between periodontitis and autoimmune responses: 1. Presence of microorganisms (commensal or pathogenic) that leads to constant antigenic stimulation by microbe-associated molecular patterns (MAMPs) (223); 2. Prolonged presence of immune activation by damage-associated molecular patterns (DAMPs) (224), given the persistent production of “damage” with a large generation of residues; 3. Constant activation of the innate and acquired immune response, including regulatory mechanisms; and 4. Continuous change in the characteristics of the environment influenced by external factors, including constant passage of microorganisms through the mouth and lifestyles, that trigger the need for uninterrupted epigenetic adaptation (225).

On the other hand, the relatively low prevalence of severe periodontal damage in the population highlight the importance of genetics, mediating not only susceptibility to infection (226), but differences in host response (227) and even susceptibility to the occurrence of inflammatory diseases (228). The 2009-2012 NHANES identified severe periodontitis in 8.9% of US adults (229) and an average worldwide prevalence of severe periodontitis has been estimated to be 11%, including countries with relatively little emphasis on periodontal health care (230). Despite the low prevalence of severe disease, the need to control dental biofilm as the main risk factor in the occurrence of periodontitis should be emphasized, as it has been found that fair to poor oral hygiene increases the risk of periodontitis by two‐ to five‐fold and that the effect of oral hygiene on periodontitis is stronger than those of other risk factors, such as diabetes, smoking or obesity (231).

Autoimmune responses have been described in both periodontally healthy and diseased individuals; but is in susceptible individuals where it is observed that the inflammatory process ultimately results in damage, with a constant altered regulation of the local response. From the data collected, the following assumptions were made based on the analysis of the autoimmune mechanisms occurrence in periodontitis:

Neither the autoimmune mechanisms dependent on the microorganism or those caused by activation and local dysregulation of immune responses by dysbiosis, can explain the occurrence of tissue damage in periodontitis; the studies as presented just support their activation. Superantigens and inhibitory receptors inactivation could not be shown to be related to the occurrence of tissue damage due to autoimmunity in periodontitis; others such as bystander activation that ends in the possible alteration of cytokine production are not clear, as it could eventually be linked to intracellular invasion by periodontopathic bacteria that presents more as an evasion mechanism to generate nutrients for its survival, than to an autoimmune response (232).

Similarly, the role of oral dysbiosis in the induction of the inflammatory response mediated by cytokine hyperproduction is not yet clear, especially since the mechanisms have been proposed from autoimmune diseases as an explanation for the damage caused by the inflammatory response itself (15). However, this does not seem to be the case in periodontitis, since the few studies that use *P. gingivalis* for disease modeling show dual functions for the main cytokines with inflammatory functions in autoimmunity, eg. the hyperproduction of IL-17 of great interest in the pathogenesis of periodontitis due to its involvement both in the inflammatory response and in the protective immunity against microorganisms (233). However, this makes it a double-edged sword in a disease like periodontitis that is initiated by the bacteria, and in which tissue damage is done and controlled by the host’s response induced by the microorganism and not by autoantigens since the autoimmune mechanisms in periodontitis seem to be part of the physiological repair process.

In relation to the above and under the exposed premise that autoimmune mechanisms overlap, the role of TLRs dysregulation in autoimmune responses in periodontal disease is controversial and complicated, since there appears to be potential heterogeneity of responses through TLRs mediated by different commensals (150, 234). Thus, the activation of certain TLRs leads to the activation and that of others to the inhibition of the differentiation of cell populations related to autoimmune responses, especially Th17 (235).

The mechanism by which spreaders, such as Pep19, direct the epitope spreading toward autoantigen formation in chronic periodontitis or in experimental periodontitis induced by *P. gingivalis* (175) is unclear. What is deduced from the studies, both in animal model and in humans, is that the response to the spreader is found in healthy individuals and is maintained without being replaced by responses to other epitopes, in chronic periodontitis and even in autoimmunity (87, 176).

Regarding the role of autoantigen complementarity and the increased auto-antibody response found in periodontitis, they have traditionally been proposed as autoimmune responses involved in tissue damage; but in light of new concepts on host-microorganism interactions and microbial dysbiosis in this disease (236), the tissue damage associated with its presence in inflamed tissues occurs more due to the dysregulation of physiological mechanisms guided by a dysbiotic inflammophilic microbiota, and is needed to cover the removal requirements of apoptotic bodies and tissue degradation proteins. The presence of some autoantibodies, in gingival fluid and serum show similar specificity in healthy individuals and periodontitis patients (194), suggesting that autoantibodies are a normal part of the immune repertoire. Therefore, the presence of autoantibodies in periodontitis does not support their causal role in the occurrence of local tissue damage (**Figure 4**).

In conclusion, in periodontitis, additional consideration should be given not only to the presence or absence of cells with autoimmune functions but to whether or not they are activated and directly related to tissue damage. Despite the lack of evidence to support some autoimmunity mechanisms in periodontitis (which does not mean that they do not participate in the pathogenesis), and after analyzing the physiological functions of such mechanisms in other inflammatory and autoimmune diseases, we hypothesize that the presence of autoreactive cells in periodontal tissues do not explain their direct relationship with tissue loss and may represent the activation of damage “control” mechanisms, as supported by the evidence of activation of the same mechanisms in periodontally healthy patients.

However, the translocation of oral microorganisms, its components or its metabolites from periodontal tissues could be involved in the occurrence of autoimmune responses at a systemic level, in which the different mechanisms mentioned are represented, even if the dynamics exact of this relationship have not yet been clarified. Thus, at systemic level, the link of autoimmunity and periodontopathogens needs to be interpreted from the role of dysbiosis in the occurrence of systemic autoimmune responses, related to the possibility of periodontopathic bacteria ability to generate an alteration of the microflora in other organs with the consequent activation of autoimmunity mechanisms. The processes by which the presence of oral pathogens in remote sites generate dysbiosis in different organs, which in turn dysregulate the systemic immune response in individuals susceptible to autoimmunity, are just being clarified.

## Author Contributions

All authors contributed to the article and approved the submitted version.

## Conflict of Interest

The authors declare that the research was conducted in the absence of any commercial or financial relationships that could be construed as a potential conflict of interest.

## Abbreviations

aCL, Anti-cardiolipin antibodies; ACPA, Anti-citrullinated protein antibodies; APCs, Antigen presenting cells; ANCAs, Anti-neutrophil cytoplasmatic antibodies; oxLDL, Anti-oxidized LDL; CTL4, cytotoxic T lymphocyte – associated protein 4; DNA, Deoxyribonucleic acid; GAD65, Enzyme glutamate decarboxylase; HSP, Heat shock proteins; IgG, Immunoglobulin G; IR, Inhibitory receptors; IFN-γ, Interferon gamma; IL, Interleukin; LPS, Lipopolysaccharides; MMPs, Matrix metalloproteinases; MS, Multiple sclerosis; MDSCs, Myeloid derived suppressor cells; NETs, Neutrophil extracellular traps; ox-LDL, Oxidized low-density lipoprotein; PRR, Pattern recognition receptor; PD-1, programmed cell death protein 1; PADs, Peptidyl-arginine deiminase; PR3, Proteinase 3; REGA, Remnant epitopes generate autoimmunity; RA, Rheumatoid arthritis; SLE, Systemic lupus erythematosus; TCR, T cell receptor; Th17, T helper 17 cells; TGF-β, Transforming growth factor beta; TNF-α, Tumor necrosis factor-α; V, Variable region.
